# Alteration of Lipid Metabolism in Prostate Cancer: Multifaceted Oncologic Implications

**DOI:** 10.3390/ijms24021391

**Published:** 2023-01-11

**Authors:** Milica Zeković, Uros Bumbaširević, Marko Živković, Tomislav Pejčić

**Affiliations:** 1 Centre of Research Excellence in Nutrition and Metabolism, Institute for Medical Research, National Institute of Republic of Serbia, University of Belgrade, 11000 Belgrade, Serbia; 2 Clinic of Urology, University Clinical Center of Serbia, 11000 Belgrade, Serbia; 3 Faculty of Medicine, University of Belgrade, 11000 Belgrade, Serbia

**Keywords:** prostate cancer, lipid metabolism, lipogenesis, fatty acids, urologic oncology

## Abstract

Cancer is increasingly recognized as an extraordinarily heterogeneous disease featuring an intricate mutational landscape and vast intra- and intertumor variability on both genetic and phenotypic levels. Prostate cancer (PCa) is the second most prevalent malignant disease among men worldwide. A single metabolic program cannot epitomize the perplexing reprogramming of tumor metabolism needed to sustain the stemness of neoplastic cells and their prominent energy-consuming functional properties, such as intensive proliferation, uncontrolled growth, migration, and invasion. In cancerous tissue, lipids provide the structural integrity of biological membranes, supply energy, influence the regulation of redox homeostasis, contribute to plasticity, angiogenesis and microenvironment reshaping, mediate the modulation of the inflammatory response, and operate as signaling messengers, i.e., lipid mediators affecting myriad processes relevant for the development of the neoplasia. Comprehensive elucidation of the lipid metabolism alterations in PCa, the underlying regulatory mechanisms, and their implications in tumorigenesis and the progression of the disease are gaining growing research interest in the contemporary urologic oncology. Delineation of the unique metabolic signature of the PCa featuring major aberrant pathways including de novo lipogenesis, lipid uptake, storage and compositional reprogramming may provide novel, exciting, and promising avenues for improving diagnosis, risk stratification, and clinical management of such a complex and heterogeneous pathology.

## 1. Introduction: Metabolic Reprogramming as a Hallmark of Malignancy

Cancer is increasingly recognized as an extraordinarily heterogeneous disease featuring an intricate mutational landscape and vast intra- and intertumor variability on both genetic and phenotypic levels. The complexity of cancer is reflected in a high level of diversity in terms of the underlying mechanisms and drivers of carcinogenesis, manifestation and progression of the disease, spectrum of metabolic and functional modifications of the affected tissue, variety of cellular processes and involved molecular actors, an array of disrupted regulatory circuits, and, finally, clinical outcomes. Furthermore, cancer represents a dynamic system that evolves over the course of qualitatively distinct states regulated by a wide range of entities operating on multiple spatial and temporal scales [1,2,3].

Under physiological conditions within intricate hierarchical systems, cells receive, send, and process sets of signals that condition the precise coordination of their behavior with the aim of preserving the stability and integrity of a complex structure such as a multicellular organism. Following comprehensive communication and control mechanisms, cells differentiate, grow, divide, and, finally, die when that serves the organism’s well-being. The formation of a malignant phenotype is due to changes that affect the control systems of cell proliferation, senescence and longevity, relationships with other cells, and capacities to evade the surveillance and defense activities of the immune system. The interplay between genetic alterations and environmental factors modulates the expression of both oncogenes and tumor-suppressors, thus orchestrating the nonlinear progression of the neoplastic disease [4]. With uncontrolled growth, cancer disturbs the normal development of physiological functions and the structural integrity of tissues, causing different specific manifestations of the disease depending on the localization, histopathologic composition, and dimensions of the tumor mass.

Conceptualization of the set of shared biological attributes of malignantly altered cells represents a theoretical framework for distilling the complexity of neoplasia as well asa heuristic tool for research and a better understanding of cancer biology. Key hallmarks of cancer cells are the maintenance of proliferative signaling, avoidance of exogenous growth suppressors, resistance to mechanisms of programmed cell death, replicative immortality, induction of angiogenesis, ability to invade and colonize distant sites reserved for other cells, alteration of cellular bioenergetics, i.e., metabolism reprogramming, and evasion of immune system-mediated destruction [5]. These acquired functional capabilities, although interdependent, act complementary and synergistically, enabling the formation, growth, and metastatic dissemination of tumors.

There is a mounting body of evidence underpinning the significance of malignancy type and tissue-specific metabolic alterations, fluctuations in bioenergetic sources, and oxidative stress modulation in supporting the anabolic requirements of tumor biomass production and cancer progression. It is evident that a single metabolic program cannot epitomize the perplexing reprogramming of tumor metabolism needed to sustain the stemness of neoplastic cells and their prominent energy-consuming functional properties, such as intensive proliferation, uncontrolled growth, migration, and invasion [6]. Each cell-cycle passage requires appropriate precursors, energy, and biosynthetic activity to duplicate biomass components in daughter cell generation, thus posing a profound metabolic challenge. Major reorganization of signal transduction pathways and rewiring of transcriptional networks creates a platform that accommodates the massive metabolic demand of rapidly proliferating malignant tissue [7]. Most prominent alternations of cancers’ cellular bioenergetics comprise the intensification of glycolysis, elevation of the glutaminolytic flux, upregulation of macromolecule biosynthesis, induction of both oxidative and nonoxidative pentose phosphate pathways, amplification of mitochondrial biogenesis, and elevation of lipid metabolism [8].

Most solid tumors exert the so-called Warburg Effect, a phenomenon described in the mid-19th century with the pioneering works by Otto Warburg, the Nobel Prize winner, whereby cancer cells take up glucose at substantially higher rates than the surrounding non-transformed normal tissue and shift their dominant adenosine triphosphate (ATP)-producing pathway towards aerobic glycolysis. In such circumstances, even under normoxia and with completely functioning mitochondria, the glucose-derived carbon is preferentially converted to lactate rather than being oxidized as pyruvate within the tricarboxylic acid cycle (TCA) [9]. Despite copious scientific endeavors and several explanatory proposals emerging over the years, definitive drivers and biological rationales behind aerobic glycolysis, its ontology, and association with proliferation in cancer remain elusive. Per unit of glucose consumed, aerobic glycolysis is a less efficient trajectory of generating ATP compared to the energy obtained via mitochondrial respiration. However, if the glycolytic influx remains high enough, due to inherent kinetic differences, the yield of synthesized ATP is comparable to oxidative phosphorylation [10]. It has been hypothesized that the Warburg Effect represents an adaptation mechanism in whichthe increased glucose consumption provides biosynthetic precursors of branching anabolic processes emanating from glycolysis. In this setting, due to the metabolic requirements of intensively proliferating cells that extend beyond ATP, the excess carbon is diverted towards de novo production of cellular building blocks, i.e., lipids, amino acids, and nucleotides needed to generate novel biomass [11]. Furthermore, the high lactate output acidifies the microenvironment, thus creating a growth advantage for cancer cells with resistant phenotypes while other cells deteriorate [12].

Nevertheless, aerobic glycolysis is only one element of the cancer-related metabolic reprogramming puzzle. To engage in limitless replicative division, cancer cells require a continuous supply of both glucose and glutamine, representing the principal and most abundant extracellular nutrients, for the metabolic pathways governing the synthesis of the three major macromolecule classes [13].Glutamine has a pleiotropic role in cancer cell survival because it is involved in numerous energetic and signal-transduction pathways, the preservation of mitochondrial metabolism, generation of antioxidants fundamental for the maintenance of redox balance and subsequently tumor homeostasis, evasion of programmed cell death signaling, and, finally, metastatic dissemination. Due to limited pyruvate availability conditioned by aerobic glycolysis, glutamine anaplerosis predominantly drives the TCA. In a multistep process of glutaminolysis, glutamine is first converted to glutamate via glutaminase, which is subsequently transformed into alpha-ketoglutarate and fed into the TCA. Furthermore, glutamine serves as a critical nitrogen donor for the synthesis of nonessential amino acids, purines, and pyrimidines, and as a carbon donor in the synthesis of fatty acids [14]. Moreover, controlled oxidation and remodeled catabolic patterns of glucose and glutamine-based carbon skeletons enable neoplastic cells to capture their reducing potential in the form of NADH, FADH2, and NADPH, which mediate the electron-transfer in a wide array of biosynthetic reactions and contribute to the mitigation of oxidative stress damage [15].

Functionally interdependent with cancer-specific glucose and glutamine catabolic pathways, reprogramming of the lipid- and cholesterol-associated metabolisms encountered in malignancy are of exceptional importance for the pathogenesis of cancer. Although their relevance was disregarded in the past, they now represent a topic of burgeoning scientific interest. Cancer cells exert pronounced avidity for lipids, which are derived endogenously from citrate or taken up from exogenous sources. In normal adult mammalian tissues, de novo lipogenesis is rather low with the exception of lipogenic tissues such as liver, adipocytes, and mammary epithelium during lactation. Conversely, tumorigenesis is associated with a remarkable amplification of lipid production [16]. Excessive lipids in cancer cells are stored in cytoplasmic multifunctional, dynamic, lipid-enriched organelles denominated as lipid droplets (LDs). Contemporary advances in lipidomic detection and analysis imply that these cell structures may not only serve in lipid storage and trafficking, but also partake in several processes associated with hallmarks of cancer. Furthermore, mounting evidence suggests an association between the increased accumulation of LDs and cancer aggressiveness [17]. In cancerous tissue, lipids provide the structural integrity of biological membranes, supply energy, influence the regulation of redox homeostasis, contribute to plasticity, angiogenesis and microenvironment reshaping, mediate the modulation of the inflammatory response via metabolic competition, exosomes and oncometabolites, and operate as signaling messengers, i.e., lipid mediators affecting myriad processes relevant for the development and progression of oncopathologies [4,18].

Contrary to their normal counterparts, whose survival and proliferation are dependent on specific signaling prerequisites based on adhesion and growth factors, cancer cells foster oncogenic alterations to circumvent dependence on these external inputs. Several aberrant oncoproteins and tumor suppressors, such as phosphoinositide 3-kinase (PI3K)/Akt/mammalian target of rapamycin (mTOR)signaling pathway, hypoxia-inducible factor-1 alpha (HIF-1A), MYC, and p53, have been associated with the regulation of the metabolic adaptation that favors tumorigenesis by facilitating cellular proliferation, access to vasculature, and stress resistance [19]. The modern approach in cancer research implies a shift from the traditional “cancer cell-centered” concept to a more comprehensive paradigm that places neoplastic cells in a complex network of interstitial extracellular matrices and stromal cells, including fibroblasts, adipocytes, mesenchymal, vascular, and immune system cells, which together form the tumor microenvironment (TME). The plastic and dynamic ecosystem of a TME is usually characterized by poor or dysfunctional vasculature, causing nutrient scarcity as well as limitations in oxygen delivery and unreliable waste removal [20]. Due to metabolism modification, the microenvironment of solid tumors undergoes a plethora of consequential changes toward a hostile milieu featuring hypoxia and acidosis [21]. Unlike normal cells, which adapt the proliferation rate and modulate the balance between the anabolic and catabolic pathways in reaction to fluctuations in bio-fuel availability, neoplastic cells continuously exhibit uncontrolled growth even under nutrient paucity and oxygen deprivation. Illuminating the reciprocal metabolic cross-talk and bidirectional interaction between cancer and diverse cellular populations and a cellular components of TME may provide better comprehension of neoplastic transformation, tumor progression, and even resistance to tumoricidal agents [22].

## 2. Prostate Cancer Overview–Epidemiology, Pathophysiology, and Treatment

Prostate cancer (PCa) is the second-most prevalent malignant disease among men worldwide [23]. The incidence of PCa varies between different geographic regions, with the highest rates observed in countries with a high human development index. On the contrary, higher mortality rates were found in less developed countries, with PCa being the fifth-leading cancer-related cause of death. Variations in incidence and mortality could not be attributed to the practice of prostate-specific antigen (PSA) screening only, but also to nonmodifiable, environmental, and lifestyle factors. In the vast majority of men, PCa is an indolent or slow-growing disease, whereas in some patients, an aggressive form is found. Given clinical heterogeneity, risk factors could be differentiated for all PCa cases and for advanced or fatal disease [24]. Well-established risk factors for PCa in general are: older age, African-American descent, and family history. Older age is the single most significant risk factor for the occurrence of prostate cancer, as after 55 years of age, the incidence increases exponentially [23,25]. Differences in the incidence of PCa are observed among racial and ethnic groups. The highest incidence and mortality rates are found in Black men, though there is disagreement over if those findings represent disparity in access to care or genetics [26,27]. Strong evidence suggests that men with PCa-positive family histories are both more likely to be diagnosed with PCa and to die from it [28,29]. Genome-wide association studies (GWAS) provided emerging evidence of genetic predisposition to PCa, confirming more than 180 genetic risk loci [30,31]. The relationships of risk factors to advanced or fatal PCa were further investigated with more or less conflicting results. Obesity is associated with a higher risk of death and biochemical recurrence of PCa, with weight loss after PCa diagnosis suggesting better prognosis [32,33]. Height is another risk factor for both overall and advanced PCa. It has been demonstrated that with every 10 cm of height, risk for developing advanced PCa also increases. The association of height and PCa is explained with exposure to growth hormones and other factors during puberty, when the prostate reaches its maturation [34]. The risk of advanced or fatal PCa was shown to be inversely correlated with physical activity. Men who engaged in recreational physical activity had a lower risk of aggressive PCa, slower disease progression, and improved survival [35]. It has been proposed that smoking promotes carcinogenesis and modifies hormonal pathways, thus causing more aggressive PCa. The largest-scale cohort study proved that smokers had a 60% higher PCa mortality risk compared to nonsmokers. Further research is required to delineate the association between dietary patterns, specific foods, and PCa.

PCa treatment requires an individual approach to each patient, and the decision is made depending on the stage of the disease while respecting the wishes and preferences of the patient. Active surveillance, surgery, and radiotherapy are reserved for localized PCa, with hormone therapy, chemotherapy, and target therapy being used in locally-advanced, advanced, or metastatic PCa.

## 3. Alterations of Lipid Metabolism in Prostate Cancer

**Figure 1:**
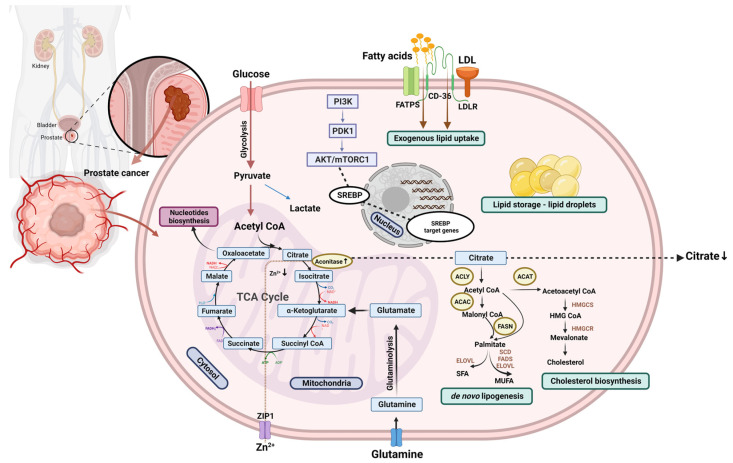
Graphical presentation of lipid reprogramming in prostate cancer. ACAC, acetyl-CoA carboxylase; ACAT, acetyl-CoA acetyltransferase; ACLY, ATP-citrate lyase; CD36, CD36 molecule; ELOVL, elongation of very long lipids protein; FASN, fatty acid synthase; FADS, fatty acid desaturase; FATPs, fatty acid transport proteins; HMGCR, 3-hydroxy-3-methylglutaryl-CoA reductase; HMGCS, 3-hydroxy-3-methylglutaryl-CoA synthase; LDL, low-density lipoprotein; LDLR, low-density lipoprotein receptor; MUFA, monounsaturated fatty acids; SCD, stearoyl-CoA desaturase; SFA, saturated fatty acids; SREBP, Sterol regulatory element binding transcription factor; TCA, tricarboxylic acid cycle.

Benign prostatic tissue exerts a specific metabolic profile in baseline circumstances. Conventionally, mammalian cells energetically depend on aerobic respiration and, therefore, rely on citrate oxidation as a crucial step in the TCA cycle. Nevertheless, one of the major biochemical functional features of the glandular epithelium in the peripheral zone of the prostate is its exceptionally high citrate production and its subsequent secretion into the prostatic fluid. The unique metabolic programming of benign prostate cells to produce rather than oxidize citrate is biologically determined by another extraordinary feature: their capability to accumulate large intracellular concentrations of zinc. Increased zinc levels exhibit an inhibitory effect on the activity of m-aconitase, the mitochondrial enzyme catalyzing the stereospecific citrate conversion to isocitrate within the Krebs cycle. In other cell types, such inhibition is lethal, but in healthy prostate tissue, it enables secretion of citrate into the semen, where it serves as the buffering agent and energy source for spermatozoa as well as afree radical scavenger and chelating agent for zinc and calcium ions [36,37]. By conforming to zinc-accumulating, citrate-producing, bioenergetically demanding metabolic phenotypes, benign prostate epithelial cells appear to halt the TCA cycle and evade dependence on the oxidative phosphorylation for acquiring energy, and thus naturally resort to aerobic glycolysis [38]. Intriguingly, research findings on metabolic aberrations in prostate malignancy indicate the significant dysregulation of the zinc–citrate homeostasis manifested with a marked decrease in their cellular concentrations compared to normal tissue or benign hyperplasia [39]. Such alterations have been consistently observed irrespective of the research diversity regarding patient population structure, clinical settings, prostate cancer stage, tissue sampling procedures, analytical assay methods, and heterogeneity of other relevant variables. Reduction of the citrate level, documented in both ex vivo and in vivo conditions with several specialized analytical techniques, including magnetic resonance spectroscopy, mass spectroscopy and desorption electrospray ionization mass spectrometric imaging, has been acknowledged as one of the most prominent, specific, and reliable malignant loci indicators in PCa [40,41,42]. Given that a uniquely high zinc concentration is the causative factor of citrate accumulation and production, its decrease precedes the decline in citrate levels. This occurs in the premalignant stage, i.e., prior to the overt histopathological evidence of malignancy, presumably due to downregulation of the ZIP1 plasma membrane uptake transporter [43,44]. Less zinc enables m-aconitase to recuperate its activity and permits the citrate oxidation further in the TCA cycle, leading to additional ATP production and enhancement of glandular-cell energetic efficacy. Furthermore, the depletion of zinc in malignant cells counteracts its tumor-suppressing effects related to apoptotic regulation and inhibition of specific pathways mediating the invasive, angiogenic, and metastatic potential of the developing neoplasia. This “genetic/metabolic” transformation of the neoplastic cells in the prostate tissue is driven by the requirements of their generational propagation and further progress of the malignant process [45] (Figure 1). Contrasting with the majority of solid tumors, the PCa pathogenesis does not adhere to the principles of the Warburg effect, as these cells do not appear to have the prerequisite elevated glucose uptake. It is noteworthy that this has particular clinical relevance, as the positron emission tomography (PET) imaging employing ^18^F-fluorodeoxyglucose (FDG) cannot provide accurate differentiation between healthy and cancerous prostates [46].

In order to support proliferation concomitantly with the shift toward citrate-oxidizing metabolism, malignant prostate cells display the lipogenic phenotype. As opposed to most normal somatic cells, which predominantly utilize the lipids from exogenous sources, de novo fatty acid biosynthesis is reported to be exacerbated in various cancer cells irrespective of the abundance of the extracellular lipid content and circulating fatty acids [47]. The significant de novo lipid production has been observed in the early stages of PCa and is further intensified as the disease progresses toward metastatic, castration-resistant PCa (mCRPC). The large amount of citrate secreted to the prostatic fluid under normal conditions not only serves as an intermediate in the TCA cyclein PCa, but also as a substrate for lipogenesis and cholesterogenesis [48]. Citrate is typically produced in the mitochondria of mammalian cells, where it either undergoes oxidation through the TCA cycle or is exported to the cytosol where ATP citrate lyase (ACLY) catalyzes its conversion to oxaloacetate (OAA), which is critical for the sustained production of aspartate partaking in the nucleotide and polyamine synthesis, and acetyl-coenzyme A (acetyl-CoA), a pivotal precursor of lipid production [49]. In healthy prostate cells, as previously elaborated, a minor amount of citrate is oxidized as it is primarily destined to be secreted to the seminal fluid. Therefore, it is reasonable to suppose that the activity of ACLY in normal prostate tissue is quite poor or absent. Malignant aberration leads to upregulation of the ACLY and inapt activity of lipogenesis in the prostate‘s ordinarily non-lipogenic tissue, thus conferring the growth and adaptive advantage to cancer cells. In addition to ACLY, increased expression of a repertoire of other lipogenic enzymes has been confirmed within PCa metabolic aberration. Such transformation is putatively facilitated by stimulation via pro-oncogenic signaling pathways such as phosphatidylinositol-3-kinase (PI3K)/ protein kinase B (PKB/AKT), Ras/extracellular signal-regulated kinase (ERK), BRAF, and HER2 [18,50]. Coordinated sequential activity of three cytosolic enzymes is warranted to generate palmitic acid. As noted, ACLY cleaves citrate to yield 2-carbon acetyl-CoA, which is then a substrate for acetyl-CoA carboxylases (ACACs). In the next step, the irreversible carboxylation leads to malonyl-CoA, andfatty acid synthase (FASN), which ultimately catalyzes the NADPH-dependent condensation of acetyl-CoA as a primer and seven malonyl-CoA, producing the saturated 16-carbon fatty acid palmitate which may be further elongated by elongases and desaturated by stearoyl-CoA desaturases [51]. Upregulation of FASN, the leading rate-limiting homodimeric multienzyme responsible for the final catalytic step in the fatty acid synthesis, represents ubiquitous phenotypic alteration in human malignancies, including PCa [52]. Endogenously-synthesized fatty acids are subsequently modified to generate an assortment of lipids, such as phospholipids and sphingolipids, utilized to fuel the plasma membrane’s biogenesis in intensively proliferating neoplastic tissue, triacylglycerols stored in LD, or lipid mediators acting as oncogenic signaling molecules driving the maintenance of the malignant phenotype and disease progression [53]. While preventing the risk of free fatty acid-induced lipotoxicity, the intracellular reservoir of LD provides a convenient and effective energy deposit as well as a supply of building blocks for the nutrient-demanding conditions of metabolic stress encountered over PCa’s progression and metastatic dissemination [54]. Given that the neoplasia-driven adaptive alteration of lipid metabolism is highly dynamic and driven by intricate regulatory networks, scientific approaches based on integration of multi-omics datasets have unraveled unprecedented multidimensional insights into complex PCa oncobiological systems [55]. The regulation of lipid homeostasis and modulation of endogenous cholesterogenesis and lipogenesis occurs dominantly at the transcriptional level through the activation of master regulators of lipid biosynthesis, i.e., via sterol regulatory element-binding proteins (SREBPs). The human genome encodes three SREBP transcription factor isoforms: SREBP1a and SREBP1c, derived from the alternate splicing of SREBPF1, and SREBP2, encoded by the SREBPF2 gene. Although belonging to the basic helix-loop-helix–leucine zipper (bHLH-Zip) cluster of transcription factors, they are synthesized as endoplasmic reticulum (ER)-bound inactive precursors, and, in order to reach the nucleus and exert their role, the N-terminal fragment must be released proteolytically. When expressed at normal levels, SREBP-1a acts as a potent modulator of all SREBP-responding genes, andSREBP-2 preferentially activates genes encoding the cascade of enzymes required for cholesterol metabolism, including HMG-CoA synthase, HMG-CoA reductase, farnesyl diphosphate synthase, and squalene synthase, whereas SREBP-1c favors the fatty acid and triglyceride pathway by inducing the ACLY, ACAC, FASN, and the elongase complex. Nevertheless, when SREBP expression surpasses physiological levels, all three isoforms may induce the transcription of the whole array of genes featuring specific sterol regulatory elements (SREs) (Figure 1) [56,57].

There is a complex interplay between the androgens and the modulation of the lipogenic program. In neoplastic prostate cells, androgen-regulated gene expression operates via common transcription factors of several genes significant for fatty acid synthesis and the cholesterol biosynthetic pathway. SREBPs have the central role in the synchronized cascade mechanism of highly coordinated androgen-mediated control of lipid metabolism in this oncopathology. It has been demonstrated that SREBP cleavage-activating protein (SCAP), compulsory for the nuclear translocation and activation of SREBP, FASN, and several lipid-modifying enzymes, including those implicated in the mevalonate-pathway, are upregulated in an androgen-dependent manner [58]. Furthermore, protein levels of SREBP1 were positively associated with the clinical Gleason grade and tumor progression towards castration resistance. SREBP–androgen interaction is bi-directional as SREBP increases the expression of androgen receptor, thus creating a self-intensifying loop which drives the perpetual gene expression of transcription factors [59,60]. Overexpression of lipid metabolic genes regulating the activity of ACLY, ACC, and FASN has been consistently correlated with the increased neoplastic cell proliferation, induction of pro-oncogenic signaling, tumor growth, worse clinicopathological features such as tumor stage, lymph-node positivity, migratory-invasive and metastatic potential, resistance to chemotherapeutics-induced apoptotic cell-death, shorter time to recurrence, reduced survival, and overall poor clinical outcome [61,62].

Signaling pathways that elicit cancer lipogenesis culminate with the formation and accumulation of LDs. Accurate, controlled, and precise synchronization between factors of lipogenesis and lipolysis defines the net amount of lipids stored intracellularly. De novo synthetized or acquired lipids in the malignant tissue are commonly incorporated in triacylglycerols and cholesterol esters, and are then deposited within LDs. Anelevated abundance of LDs is the representative characteristic of various aggressive cancers, including PCa. The final step in the biosynthesis of triacylglycerols is catalyzed by acyl-CoA:diacylglycerol acyltransferase (DGAT) enzymes; the overexpression of DGAT1 was found in PCa neoplastic cells compared to normal epithelium [17]. Furthermore, it has been demonstrated that the disruption of the equilibrium between the anabolic/catabolic ratio may be caused by upregulation of a lipogenesis enzyme, such as DGAT1, loss of prolipolytic mediators, such as adipose triglyceride lipase (ATGL) and pigment epithelium-derived factor (PEDF), also denominated as serpin F1 (SERPINF1), or both, resulting with increased intratumoral lipid content, which is associated with augmented tumor aggressiveness [63]. Accumulating evidence impliesthat lipid mobilization from the LDs may be a promising target for anti-cancer therapy.

Although the endogenous de novo lipogenesis is considered to be the dominant source of fatty acids in neoplastic cells, it has been acknowledged that, under metabolic stress, cancers may adopt additional means to obtain lipids, including scavenging from the extracellular milieu. Such aberrant metabolic routs, coupled with the proficient de novoformation machinery, fuel the pervasive upregulation of lipid abundance and accommodate the increased demand of the progressing neoplasia [64]. Furthermore, there is evidence supporting the notion that diverse tumor types display metabolic plasticity regarding lipid acquisition pathways by activating both lipid synthesis and exogenous uptake in a context-dependent manner. In the circulation, free fatty acids are either bound to albumin or are esterified, mostly within lipid vesicles, i.e., very low density lipoproteins (VLDL) and chylomicrons. Fatty acid trafficking across cellular membrane predominantly occurs via a saturable protein-facilitated process. Lipoprotein lipase (LPL) is the fundamental enzyme of extracellular lipolysis and isresponsible for the liberation of fatty acids from circulating lipoprotein particles. A number of diverse proteins have been implicated in the facilitation of fatty acid uptake, and more may yet be identified, but the transmembrane channel CD36 has been recognized as a pivotal fatty acid translocase [65,66]. Evidence derived from prostate cancerous tissue and patient-derived xenograft mouse models suggest that CD36 blockades and subsequent inhibition of the mediated fatty acid uptake may provide a beneficial effect in a preclinical PCa setting and pave the way for the exploration of potential novel therapeutic strategies [67]. Although further research is warranted to explore and establish the role of lipolytic–lipogenic functional coupling in the regulation of oncogenesis and tumor progression, this represents a promising area of transdisciplinary investigation in the evolving discipline of molecular pathological epidemiology [68]. Given that transformed cells adjust the relative ratio and contribution of de novo lipogenesis and fatty acid uptake based on the availability of different lipid species extracellularly, there is a biologic rationale to postulate that LPL and CD36 may be significant protagonists at the intersection between dietary lipid composition and PCa biology (Figure 1).

In addition to lipogenesis, reprogramming of the fatty acid composition patterns was acknowledged in the cellular pool and membrane phospholipid profile. Fatty acid desaturation, catalyzed by the family of stearoyl-CoA desaturases (SCDs), and elongation, depending on the activity of elongases, may have a significant role in PCa progression. SCD1 overexpression was demonstrated on both mRNA and protein levels in specific PCa cell lines and human tissue specimens, where it correlated with a higher Gleason grade and worse clinicopathological features [69]. Furthermore, elevated expression of the ELOVL7 elongase was associated with PCa growth and survival via particular metabolic processes involving very-long-chain saturated FAs (SVLFAs) and their derivatives [70] (Figure 1).

Deregulation of cholesterol metabolism is another hallmark of the PCa pathogenesis. Featuring an amphiphilic and virtually planar structure, cholesterol molecules represent major constituents of the cellular membrane, supporting its integrity and regulating itsfluidity and permeability. In the TME, both intrinsic and extrinsic cues trigger cholesterol metabolism alternation, thus supporting carcinogenesis and suppressing the antitumoral activity of the immunological landscape [71]. PCa cells may obtain cholesterol by importing it from exogenous sources, such as lipoprotein particles and exosomes, by recruiting it from intracellular storage, i.e., LDs deposits, and via de novo cholesterol biosynthesis. In physiological conditions, in spite of fluctuations in serum levels, cholesterol homeostasis in prostate tissue remains tightly regulated in accordance with intracellular cholesterol and oxysterol concentrations and certain extracellular stimuli. Nevertheless, in PCa, cholesterol levels increase as a consequence of the disturbed homeostasis favoring cholesterogenesis (Figure 1). One of the substantially upregulated biosynthetic processes in PCa is the mevalonate pathway, which facilitates cholesterol accumulation. Cholesterol metabolism generates essential structural membrane components as well asmetabolites exerting a range of diverse biological functions, and it serves as a precursor of bile acids, vitamin D, and steroid hormones [72]. A quantitative imaging study performed with Raman spectromicroscopy on benign prostate tissue from healthy donors and on specimens representing a gamut of human prostate pathologies, including prostatitis, benign prostatic hyperplasia, and PCa (low-grade, high-grade and metastases), revealed aberrant accumulation of esterified cholesterol in LDs in high-grade PCa and metastatic clinical samples. This phenomenon has been associated with the tumor suppressors phosphatase and TENsin homolog deleted on chromosome 10 (PTEN) attenuation, PI3K/AKT pathway activation, and the following induction of the SREBP and LDL receptor (LDL-R). Furthermore, depletion of cholesterol-ester deposits notably reduced proliferation and tumor growth while suppressing cancer invasiveness in mouse xenograft models with negligible toxicity [73]. In a prospective study analyzing the cholesterol metabolism transcriptome in relation to PCa lethality, the absolute expression of the rate-limiting enyzme squalene monooxygenase (SQLE) was associated with increased histologic markers of angiogenesis, metastatic disease, and mortality [74]. These results were corroborated in another study reporting the low levels of LDL-R coupled with the SQLE overexpression in high Gleason-grade PCa samples associated with increased lethality, suggesting that advanced prostate neoplasia relieson de novo cholesterogenesis rather than transcellular uptake or esterification [75]. These findings herald the potential of employing diagnostic and treatment strategies targeting cholesterol metabolism in advanced PCa.

The lipidomics approach, involving comprehensive identification, structural characterization, and quantitation of the complex networks and pathways of cellular lipids and their interactions with other moieties in vivo, represents a platform of exceptional potential in PCa research. As a metabolome subset, ubiquitously present lipid species display multifaceted and diverse roles in both the etiology and sequelae of various pathologies. Exploration of lipid alterations in translational contexts and clinical settings serves not only to advance the fundamental comprehension of the biological processes underlying disease onset and trajectory, but also to assist in developing personalized risk-assessment models that may guide curative interventions [76]. Providing insight into lipid-driven mechanisms and anomalies arising from cancer-related perturbations to normal homeostasis, lipidome profiling may complement both diagnostic and therapeutic strategies by revealing novel lipid-based biomarkers and targetable metabolic modifications. Indisputable and significant progress in the perpetually evolving field of lipidomic technologies rekindled the appreciation of the malignancy-associated lipidome landscape for deciphering tumor biology, disease progression, therapeutic responsiveness, clinicopathological features, and overall prognosis. Integration of system-level and targeted lipidomics represents a powerful instrument for illuminating both common paradigms of prostate tissue malignancy and patient-specific features, which may be applied in the theranostic context [77]. Nevertheless, there are certain hurdles limiting the adoption of lipidomic assays as an accessible, practical, and effective tool in PCa management. Previous studies demonstrated an association between tumorigenesis and progression of PCa and aberrations in abundance and composition of various fatty acids, a range of phospholipid species, ceramides, and cholesterol metabolites, featuring diverse enzymes and multiple pathways [70,77,78,79]. Although several lipid panels have been proposed as potential biomarkers, the majority either failed to provide a strong correlation with tumor aggressiveness and metastasis or lacked discriminate power concerning benign hyperplasia [55]. Despite emerging imaging approaches and advanced analytical and statistical techniques that have enabled commendable achievements in this field, in-depth characterization of the PCa lipidomic atlas warrants further functional exploration and focused research endeavors. Furthermore, a vast discrepancy remains between research-based circumstances and clinical practice. Instrumentation, technical optimization, robust cross-validation, feasibility demonstration, and solid confirmation of practical utility and added benefit are the essential and ineluctable steps on the path of transition from the experimental and research phases to the clinical environment [80]. Due to the complexity of regulatory networks, the dynamic nature of cancer-adaptive metabolic reprogramming, and intricate interactions with other molecules, the confinement of focus to lipid phenotypes might not be sufficient. Integration of multi-omics datasets upgrades the breadth and depth of analysis, providing an unprecedented holistic perspective on oncogenesis drivers and cancer behavior and, thus, creating a promising platform for precision oncology [81].

## 4. Conclusions

In summary, comprehensive elucidation of the lipid metabolism alteration in the PCa, the underlying regulatory mechanisms, and their implications in tumorigenesis and the progression of the disease are gaining growing research interest in contemporary urologic oncology. A better understanding of the unique metabolic signature of PCa, featuring major aberrant pathways including de novo lipogenesis, lipid uptake, storage and compositional reprogramming, may provide novel, exciting, and promising avenues for improving diagnosis, risk stratification, and clinical management of such a complex and heterogeneous pathology.
